# Refeeding Syndrome Awareness among Physicians of King Abdullah Medical City in Makkah, Saudi Arabia

**DOI:** 10.3390/healthcare11060794

**Published:** 2023-03-08

**Authors:** Sara M. Bahashwan, Amjad A. Sindy, Firas Azzeh, Sarah O. Alkholy, Wafaa F. Abusudah, Hassan M. Bukhari, Elsayed H. Bakr, Walaa E. Alhassani, Bayan Tashkandi, Nouf Abdullah Alharbi, Wedad Azhar, Alaa Qadhi, Khloud Ghafouri

**Affiliations:** 1 Clinical Nutrition Administration, King Abdullah Medical City, P.O. Box 57657, Makkah 21955, Saudi Arabia; 2 Clinical Nutrition Department, Faculty of Applied Medical Sciences, Umm Al-Qura University, P.O. Box 715, Makkah 21955, Saudi Arabia; 3 Food and Nutrition Department, King Abdulaziz University, Jeddah 22254, Saudi Arabia; 4 Department of Nutrition and Food Sciences, Northern Border University, P.O. Box 1321, Arar 91431, Saudi Arabia

**Keywords:** refeeding syndrome, starvation, physicians, knowledge, Saudi Arabia, ICU, critical care

## Abstract

Background: Refeeding syndrome (RFS) is a lethal condition of metabolic disturbances that arise from the sudden switch of metabolism from a state of starvation to one of nourishment. Quick recognition would reduce health complications. Physicians who are unaware of the syndrome will not identify and treat it. As nutritional risk is associated with the risk of RFS, physicians should be aware of it. Aim: To determine whether the physicians of King Abdullah Medical City (KAMC) in Makkah know of RFS and, if so, have skills in diagnosis and managing the syndrome. Methods: One hundred and fifty-nine physicians of KAMC were recruited in a cross-sectional study. They were asked to complete a questionnaire by face-to-face interview. The questionnaire was designed to capture physicians’ knowledge and ability to manage RFS based on the awarding of certain scoring points. Results: The level of knowledge among physicians had a significant association between knowledge and age (*p* = 0.021) and medical specialty (*p* = 0.010). Additionally, the most knowledgeable physicians were those who work in critical care (21.4%). Around 18% of physicians were not able to manage RFS. Conclusions: Lacking knowledge of RFS and how to manage it leads to critical life-threatening complications. Physicians need nutritional education to help them diagnose RFS and consult dietitians to avoid its complications.

## 1. Introduction

Disease-related malnutrition is a common condition in hospitalized patients and is associated with destructive clinical outcomes including health complications, prolonged stay in the hospital, and mortality [1]. Over 60 years ago, at the end of the Second World War, the potentially fatal consequences of replenishing severely malnourished individuals were first reported in malnourished Japanese prisoners [2]. In 1981, the term refeeding syndrome (RFS) was introduced by Weinsier and Krumdieck, and the first deaths were reported as being due to overuse of parenteral nutrition (PN) [3]. Refeeding syndrome is a life-threatening condition with a series of metabolic disturbances during the Initial phase of nutritional therapy that arises from the sudden switch of body metabolism from a state of starvation to a state of nourishment in patients who are severely malnourished or metabolically stressed due to severe illness [4,5,6].

The syndrome encompasses severe phosphate depletion, fluid imbalance, potassium, and magnesium deficiency, alters glucose metabolism and vitamin deficiency (mainly thiamine or vitamin B1), and causes cardiac failure, pulmonary edema, seizures, hemolysis, dysrhythmias, and organ dysfunction, leading to mortality in some cases. RFS can present with any source of nutritional intervention: oral, enteral, or parenteral [4,7,8].

RFS is likely to develop within the first 72 h after the beginning of nutritional therapy and progresses rapidly. Quick recognition is vitally important and requires a well-trained medical team [4]. Physicians who are unaware of the syndrome cannot identify and treat RFS. Hence, the diagnosis will not be recorded in the patient file, neither as a simple diagnosis nor as a contributing factor of death. Therefore, the awareness of the medical team of this condition is very crucial and essential. It must be well-known by every physician to ensure early diagnosis and treatment and achieve better support for preventive measures [4,9]. Cases of RFS are frequently recognized by dietitians in clinical routines due to increased awareness. However, a lack of knowledge has been observed among physicians. Since nutritional risk is associated with the risk of RFS, awareness of both situations must be improved among the physicians in daily clinical practice.

The risk factors for the development of RFS according to the National Institute for Health and Care Excellence (NICE) guidelines in 2006 include low body mass index (<18.5 kg/m^2^), unintentional weight loss within the last 3 to 6 months, starvation or little nutritional intake for more than 5 days, history of alcohol abuse, and low initial electrolyte concentrations. Risk factors may also include underlying health conditions affecting nutrients absorption, such as short bowel syndrome, bariatric surgery, and eating disorders. In addition, chronic gastrointestinal problems such as diarrhea or vomiting may increase the risk of RFS [10]. Moreover, medical therapeutic interventions such as chemotherapy, hemodialysis, and polypharmacy are associated with a high risk of RFS [4]. However, the onset of RFS may have been precipitated by intravenous glucose infusion prior to artificial nutrition support [6]. Oncology and elderly patients are more likely to develop RFS. Malnutrition is a concern in several diseases, such as surgical/critical illness (0–100%), cancer (5–80%), neurology (4–66%), respiratory disease (5–60%), gastrointestinal and liver disease (3–100%), HIV/AIDS (8–98%), and renal disease (10–72%) [11]. In the treatment process of hospitalized patients, a compromised nutritional status such as RFS is largely overlooked, particularly in those with chronic disease [12]. While between 20% and 50% of patients in the hospital acute setting are malnourished or at risk of malnutrition, it was found that only 8% receive a medical diagnosis of malnutrition during their stay in the hospital [13].

RFS symptoms are variable, unpredictable, can develop without warning, and can appear late. The symptoms of RFS result from serum electrolytes changes that affect cell membrane potential and deteriorate cell function. The most commonly observed clinical non-specific symptoms are tachycardia, tachypnea, and peripheral edema [4]. The clinical manifestations involve various clinical abnormalities, but low serum phosphate is considered as both a hallmark and a sentinel sign [5]. However, the phosphate level has limitations in patients with multiorgan failure or renal impairment [14]. Clinical degradation may rise rapidly if the cause is not identified and appropriately treated [14,15]. The pathophysiology of RFS is still ambiguous. This might explain why there is neither a specific set of signs and symptoms nor a unified definition of RFS; rather, it is a descriptive term referring to a broad spectrum of clinical and biochemical abnormalities. This often leads to overlooking, underdiagnosing, and consequently not treating RFS [4,15]. Thus, it is not surprising that the prevalence of RFS is unknown. There is a lack of robust epidemiological studies due to the absence of internationally approved diagnostic criteria for detecting RFS [9,14]. There are three essential aspects involved in dealing with RFS: early detection of patients at significant risk, applying the appropriate refeeding protocol, and close monitoring during refeeding [15].

Effective multidisciplinary collaboration can aid in preventing many of these negative outcomes. Physicians and dietitians have a key role in the promotion of preventive measures of RFS. The role of dietitians is to screen the patient’s risk of nutritional complications, assess their nutritional requirements, prescribe the optimal feeding regimen, and assist in promoting awareness of the risk and management of RFS. Meanwhile, the role of physicians is to enhance awareness of the risk of RFS prevention and management, prescribe thiamine before starting nutritional support in patients at risk of RFS, and ensure timely biochemical monitoring and electrolyte supplementation [16]. Relatively few studies have been conducted to evaluate the ability of healthcare professionals to diagnose and treat RFS based on questionnaire distribution. Both physicians and nurses declare that their ability to identify patients at high risk of RFS and their knowledge about this condition is insufficient. A study was conducted among two groups of healthcare professionals in internal medicine and gastroenterology. Gastroenterologists reported more confidence with regard to the diagnosis and management of the complications of malnutrition, but still declared that they believed their knowledge was suboptimal [17]. Barrocas, Utech, and Mitchell (2020) reported that the most frequent causes of an inadequate diagnosis of malnutrition include clinicians’ lack of knowledge of and screening for malnutrition, the lack of effective communication to coordinate the care provided, and absence of appropriate reporting of malnutrition diagnoses. When compared to well-nourished patients, hospitalized patients with malnutrition have a five-fold rise risk of mortality and a 50% chance of hospital readmission [18]. It has been observed that RFS is not well-known among head-and-neck cancer teams; this may be related to the lack of nutritional topics in medical and oncology specialist training [15]. Another study performed to measure the knowledge among physicians and fifth-year medical students of RFS showed that the vast majority of them were unaware of this metabolic condition. The authors demonstrated that 78% of participants did not diagnose the RFS or a comparable condition [9]. 

Accurate guidelines for dealing with this syndrome have long been inadequate. In brief, in at-risk patients, nutritional support should be started cautiously and in an anticipatory manner [5]. It is important to address RFS and treat it promptly. The only way to do so is by accurate diagnosis. A physician is the only clinician who is authorized to provide a medical diagnosis of malnutrition and thus must ensure that malnutrition is documented and that ongoing care is arranged with other caregivers [12]. In Saudi Arabia, no study has been conducted to measure prevalence of RFS or the awareness of RFS in healthcare professionals. Identifying the level of knowledge of RFS among physician is vital to enhance clinical outcomes. Accordingly, this study aimed to determine the awareness among the physicians of King Abdullah Medical City in Makkah about the RFS and if they had required skills to diagnosis and manage it. The hypothesis that was tested was that the physicians lack awareness about RFS in clinical practice.

## 2. Materials and Methods

**Figure 1:**
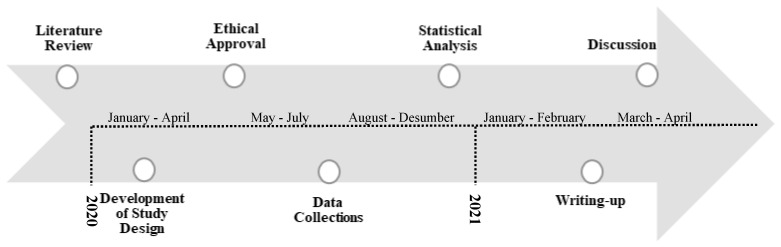
The timeline.

A cross-sectional study was conducting on physicians of King Abdullah Medical City in Makkah (KAMC). The study took place from January 2020 to April 2021 (Figure 1). Inclusion criteria were as follows: male and female consultants, assistants, and residents who are in the direct care of patients with a high risk of developing RFS (i.e., critical care unit (35 physicians), oncology center (41 physicians), neurology center (33 physicians), specialized surgery unit (40 physicians), internal medicine department, and gastroenterology department (50 physicians)). Ophthalmologists, orthopedics, dermatologists, psychiatrists, family medicine practitioners, and ENT physicians were excluded. Participation in this research was voluntary. The data eres collected face-to-face from each participant via electronic questionnaire containing direct multiple-choice questions. The questionnaire included two sections. The first section included questions about sex, age, medical position, years of professional experience, medical specialty, and if the participant has certified nutritional medicine education. The second section contained direct questions about refeeding syndrome to measure the extent of knowledge and the ability to manage the syndrome. After verification, data were directly transferred to a statistical database.

To measure knowledge, four questions were asked. A correct answer received one point, while an incorrect answer received zero. Participants were asked if they are familiar with RFS; if yes, they were asked to define RFS. The second question was about the blood values that should be checked before starting the nutritional therapy, with three correct choices: phosphate, potassium, and magnesium. Participants who scored three or four were considered to have good knowledge. Participants with low or no knowledge were provided with educational sessions about RFS and its management. To measure ability to manage RFS, twelve questions were asked. The questions were about screening patients at risk of RFS, the feeding protocol, thiamine prescription, monitoring, and the need to consult a dietitian. A correct answer received one point, while an incorrect answer received zero, so that the full score for the ability to manage RFS was twelve points. Participants who correctly answered nine to twelve questions were classified as good. Those who correctly answered five to eight questions were classified as moderate. Those who correctly answered one to four questions were classified as low, while those who did not answer any question were considered unable to manage the syndrome.

To ensure the significance and simplicity of the study questions, the questionnaire was sent to specialists, including dietitians and academic researchers. A pilot study on 15 participants was conducted to validate the questionnaire, test the simplicity of the questions, and determine the duration of the interview. Cronbach’s alpha for all tested domains (knowledge and ability to manage RFS) was more than 7, indicating acceptable reliability.

The study was approved in July 2020 by KAMC IRB, registered at the National BioMedical Ethics Committee, King Abdulaziz City for Science and Technology (Registration no.H-02-K-001), and follows the GCP-ICH regulations (OHRP Registration no. IORG0007625).

Data analysis was performed using SPSS statistical software (IBM Corp, Version 25, Armonk, NY, USA). Categorical variables are expressed as absolute numbers and relative frequencies (%). Fisher’s exact test was used for comparing the categorical variables with two levels, whereas categorical variables with more than two levels were compared by chi-squared test. One-way analysis of variance post hoc test was performed to identify significant group differences. A value of *p* ≤ 0.05 was considered significant.

## 3. Results

A total of 159 physicians from different medical positions (70.4% male) were interviewed. The majority of participants were ages 21 to 40 (65.4%). The majority of the participants (62.3%) reported professional experience of 5–20 years. The largest specialty groups of respondents were from the critical care unit (30.2%) and internal medicine (19.5%). Only 19.5% had a certified post-graduate course in nutritional medicine.

No significant relation was detected between gender, medical position, years of experience, and nutritional medical education and the knowledge of RFS. A significant association between knowledge and age (*p* = 0.021) and medical specialty (*p* = 0.010) was demonstrated (Table 1).

**Figure 2:**
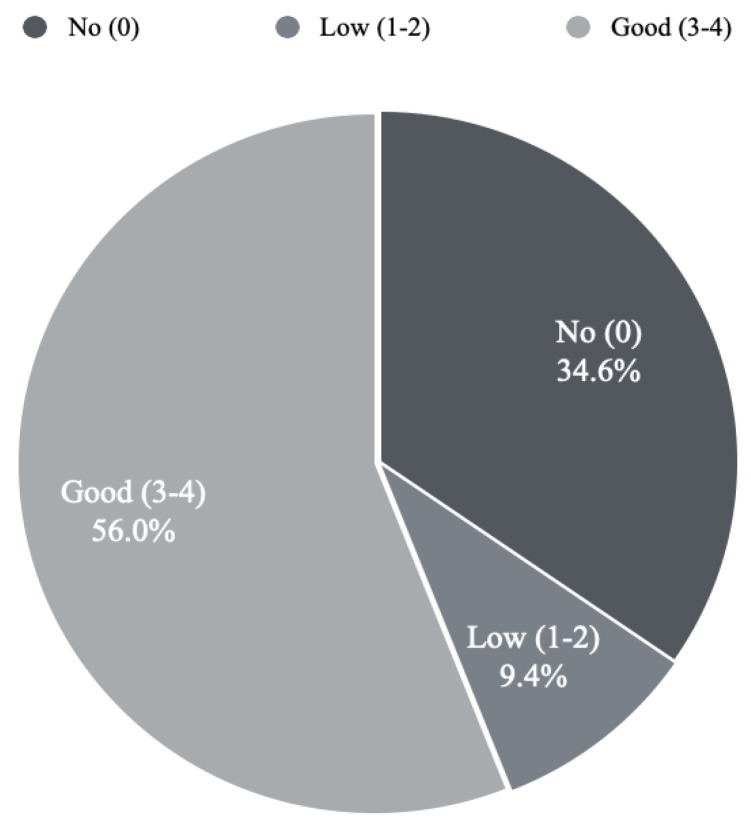
The knowledge of RFS among participants. Three levels of knowledge were measured. No knowledge, low, and good.

Of 159 participants 89 physicians (56%) scored between 3–4, demonstrating good knowledge; 15 participants scored 1–2, indicating low knowledge; and 55 (34.6%) had a score of 0, indicating no knowledge at all (Figure 2).

**Figure 3:**
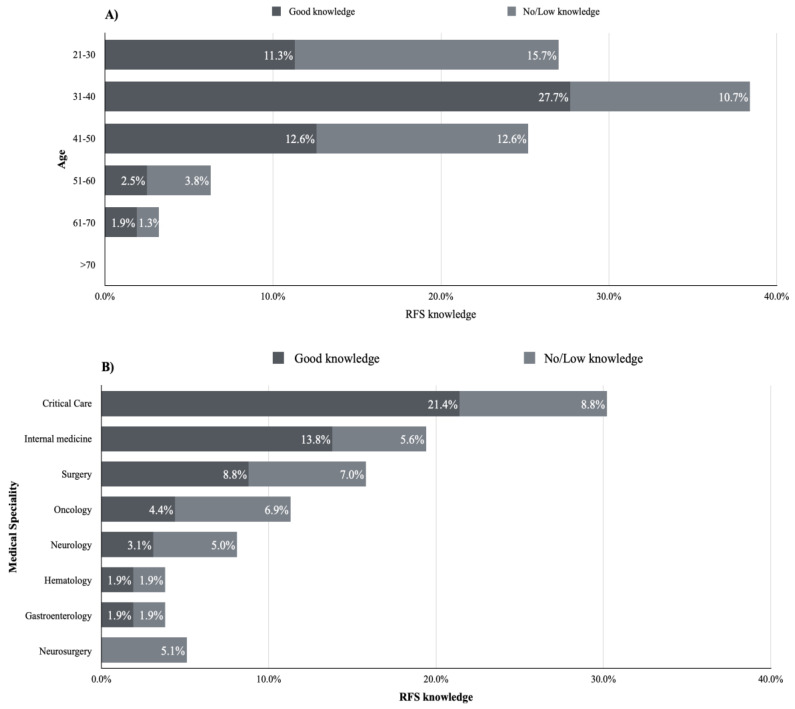
The factors influencing knowledge level among physicians at KAMC, (**A**) illustrates the influence of age on RFS knowledge, (**B**) illustrates the influence of medical specialty on RFS knowledge.

It was found that the level of knowledge increased with age (Figure 3A). However, the highest level of knowledge was observed among critical care physicians (21.4%), internal medicine physicians (13.8%), and surgeons (8.8%) (Figure 3B).

**Figure 4:**
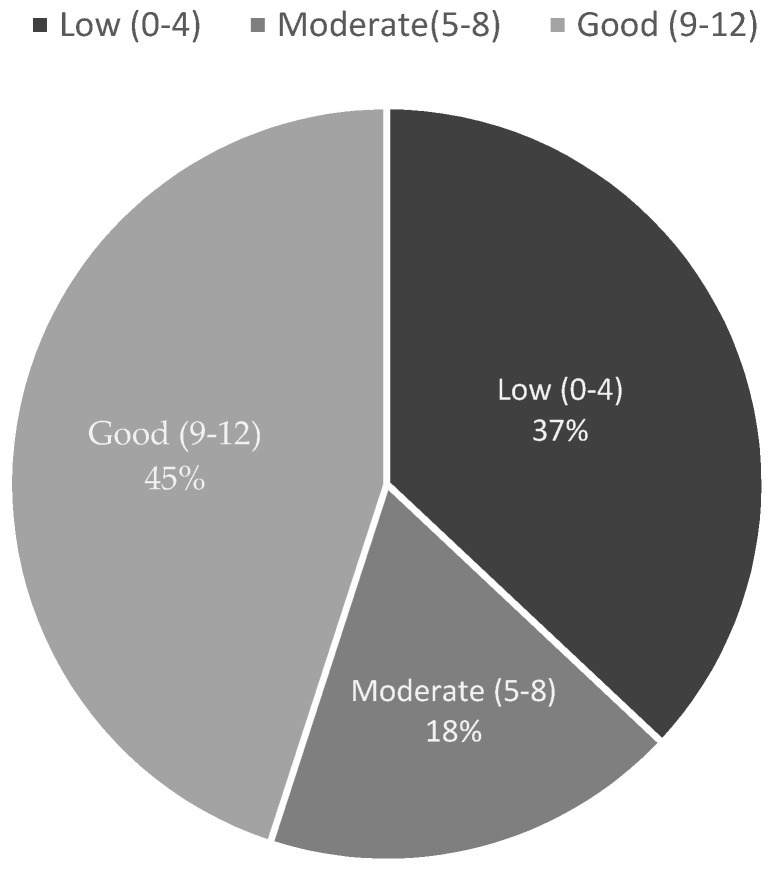
The ability to manage REF among participants.

In terms of ability to manage RFS, less than half of the physicians, i.e., 71 (45%) of them, were able to manage RFS; 18% of physicians had moderate ability, and 37% demonstrated low or inability to deal with RFS cases (Figure 4).

There was no significant relation between age, gender, years of experience, and nutritional medical education and the ability to manage RFS (Table 2). However, a significant relation was reported between the ability to manage RFS and medical position (*p* = 0.044), and medical specialty (*p* = 0.000). The rate of ability to manage RFS increased with increasing physician seniority.

There was a significant positive correlation between the knowledge and the ability to manage the syndrome *p* = 0.000. Table 3 below shows that around 41% of participants had a good knowledge as well as a good ability to deal with patients at risk of RFS, while 15.1% of them showed a lack of ability to deal with it.

The vast majority of physicians (94.3%) acknowledged the importance of consulting a dietitian for any malnourished patient (Table 4).

## 4. Discussion

The purpose of this study was to determine whether the physicians of KAMC in Makkah are aware of the syndrome and, if so, have the skills to diagnose and manage it. 

In RFS, there are numerous biochemical changes, clinical features, and complications that range from mild (asymptomatic) to severe (death). Because there is no unanimous agreement on a clear definition, physicians are less aware of the condition and overlook it [19]. In contrast with other studies, this study showed that more than half of KAMC physicians who were included in the survey demonstrated a good knowledge of RFS. This can be attributed to the significant association between knowledge and medical specialty (*p* = 0.010), as the critical care physicians and internal medicine represented 30% and 19.5% of total physicians, respectively. A possible explanation for this might be the extent to which pathological conditions are related to malnutrition, which increased the awareness of malnutrition disorders among physicians in related specialties. In a similar study, the highest rates of correct RFS diagnosis were demonstrated by internists (51%) and geriatricians (28%) [9]. The impact of RFS has been overlooked in many medical specialties, and most gastroenterologists are unconcerned about the risk of RFS during early nutritional support [20]. A retrospective multicenter study that was conducted to determine the clinical implications of RFS in patients with acute pancreatitis reported that 20.5% of patients died from acute pancreatitis within 3 days following sudden nutritional support. They demonstrated that this may explain unexpected and unexplained early mortality in acute pancreatitis patients [20].

One unanticipated finding in our study was that the majority of oncologists did not demonstrate any knowledge about RFS, even though the incidence of RFS is as high as 25% in cancer patients and mortality reaches 50% in highly developed RFS [21]. An observational study by Rasmussen et al. (2016) identified a high incidence rate of refeeding phenomena (a decline in *p*-phosphate ≥ 0.22 mmol/L) and RFS in a study population of patients with different types of head-and-neck cancers referred for surgery [22].

Our study was unable to demonstrate any significant association between gender, medical seniority, years of experience, and nutritional medical education and knowledge of RFS. In a systematic review that was conducted to identify physicians’ and nurses’ nutrition knowledge, twenty-five studies founded associations between nutrition knowledge and demographic variables such as age, gender, and educational level. Age, years of experience, holding an advanced degree, achieving nutrition education and/or training, and specializing in an area were all associated with greater nutrition knowledge. There is evidence that medical graduates around the world do not undergo adequate nutrition education [23]. In 1985, The National Academy of Sciences of the United States proposed a minimum of 25 h of nutrition education in medical schools [24]. Until 2015, only one-quarter of US professional schools met those recommendations, and similar situations have been reported in other countries. Besides the financial burden of implementing nutrition training in professional schools, another major impediment is a shortage of specialized physicians and nurses trained in nutrition [25].

Although good knowledge of RFS was found in more than half of the participants, a good ability to manage and treat the syndrome was found in only 44.6%. The key concept in the management of RFS is prevention, which requires physicians to identify this disorder in high-risk patients in the early stage and administer appropriate nutrition intervention with close monitoring [19]. In our study, there was a significant correlation between an ability for RFS management and both medical specialty and position. Almost two-thirds of critical care physicians showed a good ability to deal with RFS, while this percentage was lower in the other specialties; foor example, more than half of the internal medicine physicians did not have good ability in RFS management. By seniority, the consultants were the most adept at dealing with the syndrome, followed by specialists and then residents. Zeldman and Mary Andrade (2020) found that nutrition knowledge was lower among physicians and nurses in the areas of nutrition management for chronic diseases/conditions, metabolism of foods, and/or the macronutrients content in foods. Furthermore, higher nutrition knowledge was observed among physicians and nurses who were specialists (e.g., geriatric or cardiologists) and had more years of clinical experience [23].

Over the last two decades, an increasing number of cases addressing the problem of RFS have been reported in various clinical scenarios, such as for gastrointestinal disorders that include inflammatory bowel disease (IBD), short bowel syndrome, acute/chronic pancreatitis, liver cirrhosis, and gastrointestinal cancer. These are important digestive diseases that can cause malnutrition and potentially increase the risk of RFS by causing extended periods of fasting or significantly reduced intake. Thus, gastroenterologists should be concerned about RFS because of the high nutritional risk associated in their daily practice with gastrointestinal disorders and the common use of nutritional support in malnourished patients [26]. However, the current study found that nearly half of gastroenterologists were unaware of RFS, and most of them were unable to manage it. In accordance with these results, previous studies have demonstrated that several patients of IBD, intestinal fistula, and chronic liver disease, as well as patients who were referred for endoscopic gastrostomy due to prolonged dysphagia, have established malnutrition, and they are considered to be at high risk of RFS [26,27]. One study on patients with gastrointestinal fistulas found a 15% incidence of RFS and increased patient morbidity [28]. Therefore, gastroenterologists should be concerned about RFS when prescribing nutritional support and be proficient in its prevention, diagnosis, early intervention, and appropriate management.

Awareness of something does not necessarily translate to a good ability to deal with it: this truism is consistent with our findings. The potentially fatal nature of the RFS has been highlighted by many case reports. However, it is predominantly not recognized or perhaps inappropriately treated, particularly in the general wards [29]. The current study showed that only 41% of the participants had both a good knowledge of the syndrome and a good ability to manage it. Most of the participants showed an inability or a lack of ability to deal with it. One study evaluated the awareness of pulmonologists of the nutritional status of COPD patients and showed that routine malnutrition screening in COPD is rare among pulmonologists [30].

Recently, the COVID-19 pandemic has become a source of global suffering. COVID-19 is an infectious disease that causes an inflammatory disorder, resulting in decreased caloric intake and increased muscle catabolism. As a result, COVID-19 patients are at a high risk of malnutrition, making malnutrition prevention and nutritional management critical aspects of care. The most severe cases are observed in patients who have a chronic disease (e.g., organ failure, obesity with a body mass index of 40, type 2 diabetes, or cancers), are elderly, and/or have multiple pathologies [31,32]. According to a cross-sectional study by Li et al., 53% of elderly hospitalized patients with COVID-19 were malnourished [33]. During ICU admission, RFS is underestimated. Patients with COVID-19 are commonly at risk (old, polymorbid, undernourished, sarcopenic) and frequently ill for 9 to 15 days. They show symptoms such as fever, asthenia, a lack of appetite, reduced nutrient intake, and an energy deficit prior to being admitted to the ICU. These factors raise the risk of electrolyte imbalances (RFS) [32]. The medical and nursing care providers in charge of COVID-19 patients are frequently unaware of the negative consequences of inadequate nutrition support, particularly refeeding syndrome. As a result, every specialized unit that receives COVID-19 patients in the acute phase should have protocols in place to prevent RFS [32].

Enhancing physician awareness of the importance of nutritional assessment would aid in the early detection of malnutrition. In clinical practice, the effective assessment of malnutrition and its management face many challenges. Usually, physicians focus on the primary disease, with little attention paid to malnutrition-related diseases. This can be compounded by miscommunication within the medical team. Effective communication between physicians and dietitians can improve the recognition and management of malnutrition while also promoting healthcare quality [34]. Most of the participating consultants agreed on the importance of consulting dietitians for any malnourished patient in response to that question on the questionnaire. Physicians who disagreed were mostly residents with internal medicine specialty who had little experience and were unaware of the role of dietitians. The dietetic role is also influenced by the attitudes of other health professionals. There is agreement on the importance of dietetic management of malnutrition; nevertheless, some healthcare providers are unfamiliar with the role of dietitians roles or believe that consulting a clinical dietitian is not always worthwhile [35]. A study found that 21.3% of physicians considered the inclusion of dietitians to be an important part of their practice, while 42.1% of physicians felt that having a dietitian on staff would greatly benefit their practice, and 6.7% anticipated no benefit at all [36].

For the dietetic management of malnutrition in the elderly, Fleurke, Voskuil, and Beneken genaamd Kolmer (2020) observed that there is a dual dependency that negatively affects the role of the dietician. Dietitians rely on other healthcare professionals for screening and referrals at the start of the nutrition care process. When dietitians are called in for a consultation, the dietitian relies once again on the physicians to activate the nutritional intervention. Dietitians are caught in a bind, because a call for more consultations will strengthen the dietitian’s role while increasing reliance on other healthcare professionals. Since not all healthcare professionals are always acquainted with the role of dietitians and could have other priorities, relying on these healthcare professionals may weaken the dietitian’s role. However, dietitians can also try to take the advantage of this deficiency as positive, because a dietetic role that is not implemented in nutritional care procedures may allow the dietitian to take on a role that they believe is appropriate for good nutritional care [35].

Dietitians must realize that even with formal nutrition policies, the management of malnutrition will not always be a priority of other healthcare professionals. They also should be flexible in their role, attempting to shape it independently of these policies to provide optimal nutrition care. Furthermore, nutrition teams can help other healthcare professionals in the prevention and management of disease-related malnutrition by guiding and educating them. More work is needed to incorporate this level of expertise into routine physician treatment and referral processes.

The results of our study open several avenues for further investigation, including multidisciplinary approaches to identifying those at risk of malnutrition and providing early intervention. Furthermore, all health professionals should be required to participate in continuing nutrition education to ensure that the most recent nutrition recommendations are followed.

A key strength of the present study is that it is the first of its kind in KSA. However, one possible limitation is the bias that might emerge from the way in which the questions were asked, the personal characteristics of the interviewer, or in the respondents’ wish to give socially desirable responses. An educational session about RFS was given to physicians. However, we did not measure an increase in their awareness or improvement in their practice, which is another limitation of the study. Therefore, a further study could be conducted to see the effect of teaching sessions for physicians on RFS and the role of the dietitians in enhancing their practice.

## 5. Conclusions

RFS is potentially fatal but is preventable. Awareness of the condition is crucial in identifying patients at risk and taking measures to prevent the occurrence of and subsequent complications related to RFS. It is important that physicians pursue their medical learning to its fullest degree; but also, it is important to consider the fundamental role of dietitians on the medical team.

## Figures and Tables

**Table 1:** Characteristics of the survey participants and the knowledge of RFS (n = 159).

Variables	N (%)	No (0)	Low (1–2)	Good (3–4)	*p* Value
		(n = 55, 34.6%)	(n = 15, 9.4%)	(n = 89, 56%)	
Age:					0.02 *
21–30	43 (27%)	19 (11.9%)	6 (3.8%)	18 (11.3%)	
31–40	61 (38.4%)	13 (8.2%)	4 (2.5%)	44 (27.7%)	
41–50	40 (25.2%)	18 (11.3%)	2 (1.3%)	20 (12.6%)	
51–60	10 (6.3%)	3 (1.9%)	3 (1.9%)	4 (2.5%)	
61–70	5 (3.1%)	2 (1.3%)	-	3 (1.9%)	
>70	-	-	-	-	
Gender:					0.55
Female	47 (29.6%)	15 (9.4%)	3 (1.9%)	29 (18.2%)	
Male	112 (70.4%)	40 (25.2%)	12 (7.5%)	60 (37.7%)	
Medical Position:					0.29
Consultant	47 (29.6%)	13 (8.2%)	3 (1.9%)	31 (19.5%)	
Resident	48 (30.2%)	21 (13.2%)	6 (3.8%)	21 (13.2%)	
Specialist	64 (40.3%)	21 (13.2%)	6 (3.8%)	37 (23.3%)	
Medical Specialty:					0.01 *
Critical Care	48 (30.2%)	9 (5.7%)	5 (3.1%)	34 (21.4%)	
Gastroenterology	6 (3.8%)	2 (1.3%)	1 (.6%)	3 (1.9%)	
Hematology	6 (3.8%)	3 (1.9%)	-	3 (1.9%)	
Internal Medicine	31 (19.5%)	8 (5%)	1 (.6%)	22 (13.8%)	
Neurology	13 (8.2%)	5 (3.1%)	3 (1.9%)	5 (3.1%)	
Oncology	18 (11.3%)	11 (6.9%)	-	7 (4.4%)	
Surgery	25 (15.7%)	9 (5.7%)	2 (1.3%)	14 (8.8%)	
Neurosurgery	8 (5%)	6 (3.8%)	2 (1.3%)	-	
Other	4 (2.5%)	2 (1.3%)	1 (.6%)	1 (0.6%)	
Years of Experience:					0.14
Less than 5 years	46 (28.9%)	20 (12.6%)	6 (3.8%)	20 (12.6%)	
5–10 years	51 (32.1%)	13 (8.2%)	4 (2.5%)	34 (21.4%)	
11–20 years	48 (30.2%)	17 (10.7%)	2 (1.3%)	29 (18.2%)	
More than 20 years	14 (8.8%)	5 (3.1%)	3 (1.9%)	6 (3.8%)	
Nutritional Education:					0.76
No	128 (80.5%)	46 (28.9%)	12 (7.5%)	70 (44%)	
Yes	31 (19.5%)	9 (5.7%)	3 (1.9%)	19 (11.9%)	

* *p* ≤ 0.05.

**Table 2:** Relation between physicians’ characteristics and their ability to manage RFS.

Variables	N (%)	Low (0–4)	Moderate (5–8)	Good (9–12)	*p* Value
		(n = 59, 37.1%)	(n = 29, 18.2%)	(n = 71, 44.6%)	
Age:					0.07
21–30	43 (27%)	21 (13.2%)	11 (6.9%)	11 (6.9%)	
31–40	61 (38.4%)	15 (9.4%)	12 (7.5%)	34 (21.4%)	
41–50	40 (25.2%)	18 (11.3%)	5 (3.1%)	17 (10.7%)	
51–60	10 (6.3%)	3 (1.9%)	1 (0.6%)	6 (3.8%)	
61–70	5 (3.1%)	2 (1.3%)	0	3 (1.9%)	
>70	-	0	0	0	
Gender:					0.28
Female	47 (29.6%)	15 (9.4%)	12 (7.5%)	20 (12.6%)	
Male	112 (70.4%)	44 (27.7%)	17 (10.7%)	51 (32.1%)	
Medical Position:					0.04 *
Consultant	47 (29.6%)	13 (8.2%)	9 (5.7%)	25 (15.7%)	
Specialist	64 (40.3%)	23 (14.5%)	8 (5%)	33 (20.8%)	
Resident	48 (30.2%)	23 (14.5%)	12 (7.5%)	13 (8.2%)	
Medical Specialty:					0.00 **
Critical Care	48 (30.2%)	10 (6.3%)	5 (3.1%)	33 (20.8%)	
Gastroenterology	6 (3.8%)	2 (1.3%)	3 (1.9%)	1 (0.6%)	
Hematology	6 (3.8%)	3 (1.9%)	1 (0.6%)	2 (1.3%)	
Internal Medicine	31 (19.5%)	8 (5%)	12 (7.5%)	11 (6.9%)	
Neurology	13 (8.2%)	5 (3.1%)	2 (1.3%)	6 (3.8%)	
Oncology	18 (11.3%)	11 (6.9%)	0	7 (4.4%)	
Surgery	25 (15.7%)	7 (4.4%)	5 (3.1%)	10 (6.3%)	
Neurosurgery	8 (5%)	0	0	0	
Other	4 (2.5%)	13 (8.2%)	1 (0.6%)	1 (0.6%)	
Years of Experience:					0.09
Less than 5 years	46 (28.9%)	22 (13.8%)	11 (6.9%)	13 (8.2%)	
5–10 years	51 (32.1%)	15 (9.4%)	12 (7.5%)	24 (15.1%)	
11–20 years	48 (30.2%)	17 (10.7%)	5 (3.1%)	26 (16.4%)	
More than 20 years	14 (8.8%)	5 (3.1%)	1 (.6%)	8 (5%)	
Nutritional Education:					0.1
No	128 (80.5%)	50 (31.4%)	26 (16.4%)	52 (32.7%)	
Yes	31 (19.5%)	9 (5.7%)	3 (1.9%)	19 (11.9%)	

* *p* ≤ 0.05, ** *p* ≤ 0.005.

**Table 3:** Relation between physicians’ knowledge of RFS and their ability to manage RFS.

Items	Low (0–4)	Moderate (5–8)	Good (9–12)	*p* Value
	(n = 59, 37.1%)	(n = 29, 18.2%)	(n = 71, 44.7%)	
Knowledge				0.000 *
No (0)	55 (34.6%)	0	0	
Low (1–2)	4 (2.5%)	5 (3.1%)	6 (3.8%)	
Good (3–4)	0	24 (15.1%)	65 (40.9%)	

* *p* ≤ 0.05.

**Table 4:** Relation between physicians’ medical position and specialty and their willingness to consult a dietitian.

Items	Agree	Disagree	*p* Value
	(n = 150, 94.3%)	(n = 9, 5.7%)	
Medical Position:			0.02 *
Consultant	47 (29.6%)	0	
Resident	42 (26.4%)	6 (3.8%)	
Specialist	61 (38.4%)	3 (1.9%)	
Medical Specialty:			0.00 **
Critical Care	48 (30.2%)	0	
Gastroenterology	6 (3.8%)	0	
Hematology	6 (3.8%)	0	
Internal Medicine	24 (15.1%)	5 (3.1%)	
Neurology	13 (8.2%)	0	
Oncology	18 (11.3%)	0	
Surgery	21 (13.2%)	0	
Neurosurgery	0	0	
Other	14 (8.8%)	4 (2.5%)	

* *p* ≤ 0.05, ** *p* ≤ 0.005.

## Data Availability

The raw data supporting the conclusions of this article will be made available by the authors without undue reservation. Please get in touch with Dr. Khloud Ghafouri: at kjghafouri@uqu.edu.sa.
